# Transcriptional insights into the CD8^+^ T cell response in mono-HIV and HCV infection

**DOI:** 10.1186/s12967-020-02252-9

**Published:** 2020-02-24

**Authors:** Si-Yao Li, Zi-Ning Zhang, Yong‑Jun Jiang, Ya‑Jing Fu, Hong Shang

**Affiliations:** 1 NHC Key Laboratory of AIDS Immunology (China Medical University), Department of Laboratory Medicine, The First Affiliated Hospital of China Medical University, No 155, Nanjing North Street, Heping District, Shenyang, 110001 Liaoning China; 2 National Clinical Research Center for Laboratory Medicine, The First Affiliated Hospital of China Medical University, Shenyang, 110001 China

**Keywords:** HIV, HCV, CD8^+^ T cells, Long-term non-progressors, Resolvers, Microarray, Meta-analysis, miRNA-143-3p

## Abstract

**Background:**

Disease progression in the absence of therapy varies significantly in mono-HIV and HCV infected individuals. Virus-specific CD8^+^ T cells play an important role in restricting lentiviral replication and determining the rate of disease progression during HIV and HCV mono- and co-infection. Thus, understanding the similarities in the characteristics of CD8^+^ T cells in mono-HIV and HCV infection at the transcriptomic level contributes to the development of antiviral therapy. In this study, a meta-analysis of CD8^+^ T cell gene expression profiles derived from mono-HIV and HCV infected individuals at different stages of disease progression, was conducted to understand the common changes experienced by CD8^+^ T cells.

**Methods:**

Five microarray datasets, reporting CD8^+^ T cell mRNA expression of the mono-HIV and HCV infected patients, were retrieved from Gene Expression Omnibus (GEO). Differentially expressed genes (DEGs) were identified via integrative meta-analysis of expression data (INMEX) program. Network analysis methods were used to assess protein–protein interaction (PPI) networks, Gene Ontology (GO) terms and pathway enrichment for DEGs. MirDIP and miRDB online prediction tools were used to predict potential microRNAs (miRNAs) targeting hub genes.

**Results:**

First, we identified 625 and 154 DEGs in the CD8^+^ T cells originating from mono-HIV and HCV chronic progressor patients, respectively, compared to healthy individuals. Among them, interferon-stimulated genes (ISGs) including *ISG15, IFIT3, ILI44L, CXCL8, FPR1* and *TLR2*, were upregulated after mono-HIV and HCV infection. Pathway enrichment analysis of DEGs showed that the “cytokine–cytokine receptor interaction” and “NF-kappa B” signaling pathways were upregulated after mono-HIV and HCV infection. In addition, we identified 92 and 50 DEGs in the CD8^+^ T cells of HIV non-progressor and HCV resolver patients, respectively, compared with corresponding chronic progressors. We observed attenuated mitosis and reduced ISG expression in HIV non-progressors and HCV resolvers compared with the corresponding chronic progressors. Finally, we identified miRNA-143-3p, predicted to target both *IFIT3* in HIV and *STAT5A* in HCV infection.

**Conclusions:**

We identified DEGs and transcriptional patterns in mono-HIV and HCV infected individuals at different stages of disease progression and identified miRNA-143-3p with potential to intervene disease progression, which provides a new strategy for developing targeted therapies.

## Background

Both human immunodeficiency virus (HIV) and hepatitis C virus (HCV) infection are caused by small, highly mutable, rapidly replicating RNA viruses with the ability to establish long-term chronic pathogenic infection in human hosts. Disease progression in the absence of therapy varies significantly in mono-HIV and HCV-infected individuals. HIV-infected patients experience progressive CD4^+^ T cell loss and develop AIDS [1]. However, a small proportion of HIV-infected patients remain clinically and/or immunologically stable for years, including long-term non-progressors (LTNPs), who maintain normal CD4^+^ T cell counts for prolonged periods (> 10 years) and elite controllers (ECs), who have undetectable viremia (< 50 copies/ml) [2–4]. HCV is a major cause of chronic liver disease, cirrhosis, and hepatocellular cancer worldwide. However, a minority of people (< 30%) who resolve acute hepatitis spontaneously (HCV resolvers) [5], are characterized by undetectable HCV RNA in the presence of HCV antibodies [6]. Since HIV infection is often complicated by co-infection with HCV [7], understanding the common characteristics of the immune response in mono-HIV and HCV infection may contribute to the development of more effective therapies, aimed specifically at mono- and co-infected individuals.

Virus-specific CD8^+^ T cells play an important role in restricting lentiviral replication, and help determining the rate of disease progression in both human hosts and non-human primate models [8, 9]. During HIV infection, a close association was observed between CD8^+^ T cells targeting the HIV-Gag protein and viral control [10–13]. Elite controllers was partially linked to higher levels of cytolytic granules within HIV-specific CD8^+^ T cells [14]. Additionally, CD8^+^ T cells isolated from ECs exhibited higher polyfunctional capability in response to HIV specific antigens [15–18]. In acute HCV infection, CD8^+^ T cells have a crucial role in determining spontaneous resolution versus viral persistence. This role is clearly supported by chimpanzee studies showing that the depletion of CD8^+^ T cells hampers HCV clearance and clinical recovery [19, 20]. Cooper et al. evidenced that in contrast with chronic progressors, CD8^+^ T cells from resolvers generated more potent acute cytotoxic responses, which correlated more strongly with protection against HCV infection than the presence of anti-HCV antibodies [5]. Badr et al. demonstrated that HCV-specific polyfunctional CD8^+^ T cells in resolver patients exhibited increased proliferation and cytokine production in contrast to the cells in chronic infected individuals [21]. Comparing the transcriptional changes of the CD8^+^ T cells of mono-HIV and HCV infected individuals contributes to our understanding of the pathogenesis and immunogenicity of these viruses. Gene expression profiling has provided a unique opportunity for evaluating virus-host interactions at the transcriptional level. Several independent studies have provided useful insights into mono-HIV and HCV infection [22–25]. However, the transcriptomic profiles of CD8^+^ T cells in mono-HIV and HCV infection needs further elucidation, in order to determine any important differences or similarities.

In the current study, we used a meta-analysis approach, which aims to incorporate high-throughput data from multiple independent studies, to compare global expression profiles of CD8^+^ T cells in mono-HIV and HCV infection. Our study identified differentially expressed genes (DEGs) within the CD8^+^ T cells of a number of patient groups, including mono-HIV and HCV chronic progressors, HIV non-progressors, HCV resolvers and healthy controls. We subsequently performed a bioinformatic analysis of the identified DEGs to provide new insights into mono-HIV and HCV pathogenesis and inform the development of new therapeutic strategies for delaying disease progression.

## Materials and methods

### Microarray data collection

Expression profiling studies were identified through the Gene Expression Omnibus (GEO, http://www.ncbi.nlm.nih.gov/geo) [26]. Using the search terms “HIV AND CD8^+^ T cells” and “HCV AND CD8^+^ T cells”, nine microarray gene expression datasets, reporting the expression data for LTNPs or ECs, resolvers, chronic progressors and healthy donors were retrieved from public repositories. To reduce the heterogeneity and increase the consistency between different datasets, we selected five microarray data from Human Genome U133A or Human Genome U133 plus 2 Array (Affymetrix Company). The characteristics of the five datasets are listed in Table 1. The following information was extracted from each of the studies that were selected: GEO accession; platform; sample source and sample size. Five datasets were conducted in Affymetrix HG U133 Gene Chips and all involved CD8^+^ T cells were from PBMC. For the GSE6740 and GSE49954 datasets, chronic progressors of mono-HIV and HCV infection were labelled the “case group” while healthy donors were considered as the “control group”.

**Table 1:** Summary of the transcriptome datasets used in this study

Study	GEO accession	Platform	Sample source	Sample size
1	GSE6740	GPL96; Affymetrix Human Genome U133A Array	HIV CD8^+^ T cells	CP = 5 HD = 5
2	GSE49954	GPL570; Affymetrix Human Genome U133 Plus 2.0 Array	HCV CD8^+^ T cells	CP = 10 HD = 5
3	GSE24081	GPL3921; Affymetrix HT Human Genome U133A Array	HIV CD8^+^ T cells	NP = 24 CP = 18
4	GSE6740	GPL96; Affymetrix Human Genome U133A Array	HIV CD8^+^ T cells	NP = 5 CP = 5
5	GSE93711	GPL22931; Affymetrix Human Genome U133A Array	HCV CD8^+^ T cells	RP = 3 CP = 7
6	GSE93712	GPL22932; Affymetrix Human Genome U133A 2.0 Array	HCV CD8^+^ T cells	RP = 21 CP = 22

*HD* healthy donor, *CP* chronic progressor, *NP* non-progressor, *RP* resolver patient

For the GSE24081, GSE6740, GSE93711 and GSE93712 datasets, the HIV non-progressors and HCV resolvers were labelled as the “case group” while chronic progressors were considered as the “control group”. The HIV chronic progressors analyzed were infected at least 1 years with evident CD4+ T cell decline to < 500 cells/ul and a viral load of > 10,000 copies/ml. The HIV non-progressors analyzed were infected at least 3 years with no evidence of CD4 + T-cell decline and a viral load of < 500 copies/ml. The HCV chronic progressors and resolvers analyzed were during the acute phase of infection (≤ 36 weeks).

### Analysis of differentially expression genes (DEGs)

To find DEGs within the CD8^+^ T cells derived from mono- HIV and HCV chronic progressors compared with healthy donors, we ran “affy” [27] and “limma” [28] R packages (http://www.bioconductor.org/packages/release/bioc/html/affy.html) to assess GSE6740 and GSE49954 RAW datasets. After background correction, quantile normalization, and summarization using RMA (Robust Multichip Average) analysis by “affy” package, expression data were log2 transformed for further analysis. Empirical Bayesian model in limma was used to identify the DEGs. Significantly differentially expressed genes were defined as those with a P **< **0.05 and** ≥ **1.5-fold change cutoff.

To find DEGs within the CD8^+^ T cells of HIV non-progressors and HCV resolvers, compared with corresponding chronic progressors, the data collected from each eligible microarray study were imported into the Integrative Meta-analysis of Expression Data (INMEX) program (http://www.inmex.ca), prior to performing the meta-analysis [29]. The GSE24081 and GSE6740 or GSE93711 and GSE93712 data were annotated after converting the gene and probe IDs to the corresponding Entrez IDs. The intensity values for each probe set were log2 transformed then uploaded, processed, and annotated for data integrity. Then, batch effect correction option (ComBat) was used to reduce potential batch effect (Additional file 1) [30]. After a data integrity check, we carried out a combined P values method, which is routinely used in the meta-analysis of microarray data [29, 31]. However, in microarray meta-analysis, a larger sample size may not warrant a larger weight, as the quality of each study can be variable. Thus, we choose Fisher’s combined P values method, which offers the advantage of being a “weight-free” method. Fisher’ method could combine P-values from independent tests of significance [31]. We consider genes with a combined P value less than 0.10 cutoff as differentially expressed genes.

### Identification of DEG protein–protein interaction (PPI) networks

DEG PPI networks were analyzed using the Search Tool for the Retrieval of Interacting Genes (STRING, V10.5; http://string-db.org/) to predict gene-protein functional associations and protein–protein interactions. Subsequently, Cytoscape software (V3.5.1; http://cytoscape.org/) was applied to visualize and analyze biological networks and node degrees, after downloading analytic results of the STRING database with a confidence score > 0.4 [32].

### Gene Ontology terms and pathway enrichment

Gene Ontology (GO) and Kyoto Encyclopedia of Genes and Genomes (KEGG) pathway enrichment analysis of DEGs were performed using the Database for Annotation, Visualization and Integrated Discovery bioinformatics resources (DAVID Gene Functional Classification Tool, http://david.abcc.ncifcrf.gov/) [33]. GO terms and KEGG maps of biological functions associated with a P < 0.05 were considered to be significantly enriched.

Subsequently, we applied the microRNA Data Integration Portal (mirDIP) (http://ophid.utoronto.ca/mirDIP) [34] and the miRDB (http://mirdb.org/) [35] online prediction tools to predict potential microRNAs targeting hub genes in mono-HIV and HCV infected individuals.

## Results

### DEGs in the CD8^+^ T cells of mono-HIV and HCV chronic progressors compared with healthy donors

Firstly, we identified the DEGs in the CD8^+^ T cells of mono-HIV and HCV chronic progressors, compared to healthy donors. According to the results of our analysis, 625 genes were identified to be differentially expressed between HIV chronic progressors and healthy donors. Of the 625 DEGs, 136 genes were upregulated and 489 genes were downregulated (Additional file 2).

**Fig. 1:**
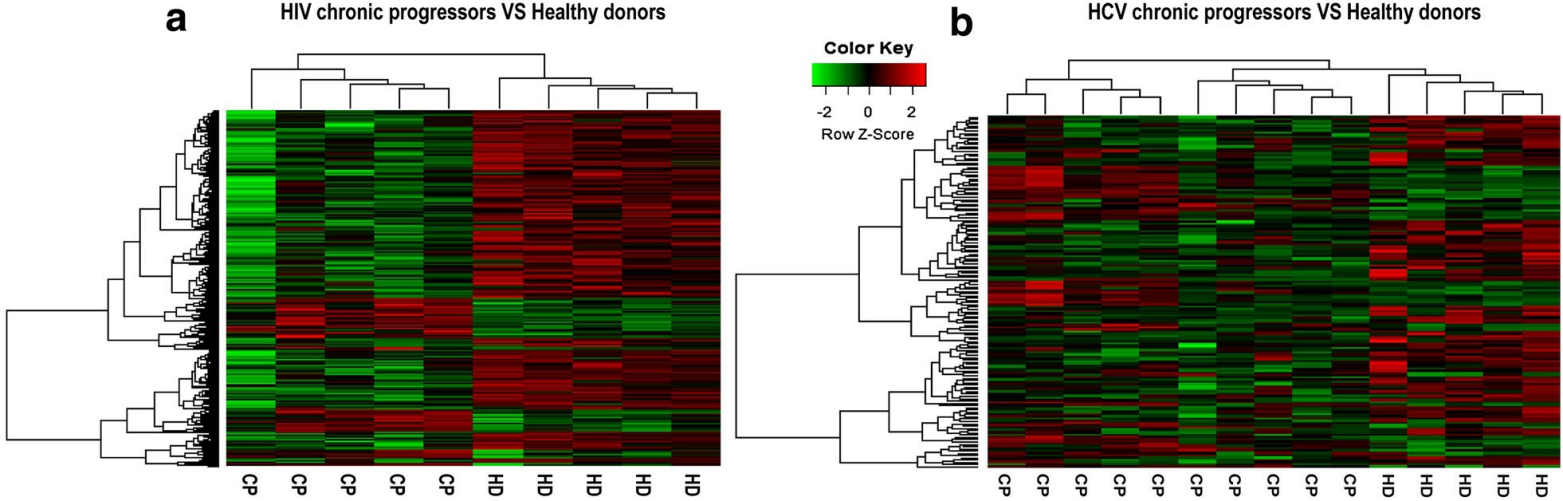
DEG heatmaps. **a** Heatmap showing DEGs from HIV chronic progressors compared with healthy donors. Each row represents a gene and each line represents a sample. Red represents higher expression and green represents lower expression. **b** Heatmap showing DEGs from HCV chronic progressors compared with healthy donors. Each row represents a gene and each line represents a sample. Red represents higher expression and green represents lower expression

154 genes, identified in the CD8^+^ T cells from HCV-infected patients, were differentially expressed between chronic progressors and healthy donors across microarray datasets. Of the 154 DEGs, 56 genes were upregulated and 98 genes were downregulated (Additional file 3). As demonstrated in the heatmap, DEGs can clearly segregate HIV (Fig. 1a) and HCV chronic progressors (Fig. 1b) from healthy donors.

### PPI network analysis of DEGs in the CD8^+^ T cells of mono-HIV and HCV chronic progressors compared with healthy donors

**Fig. 2:**
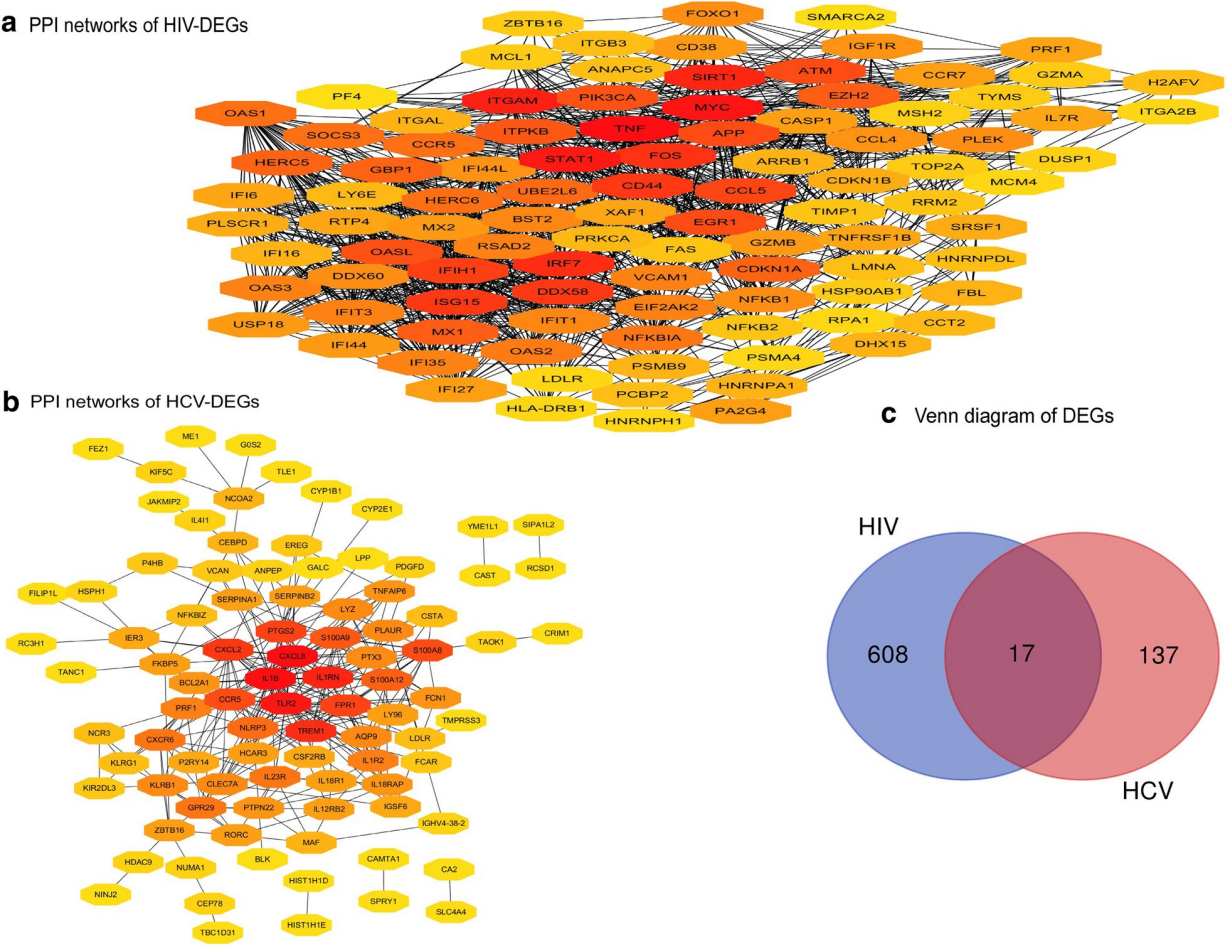
DEG PPI networks and Venn diagrams. **a** PPI network showing DEGs from HIV chronic progressors compared with healthy donors. The top 100 nodes, ranked by degree, are shown. **b** PPI network showing DEGs from HCV chronic progressors compared with healthy donors. In **a** and **b** red = greater degree, yellow = lesser degree. **c** Venn diagram of co-DEGs

Next, we performed PPI network analysis of DEGs in the CD8^+^ T cell of mono-HIV and HCV chronic progressors compared with healthy donors, to identify the hub nodes. We identified 542 nodes from the PPI network of the CD8^+^ T cell-specific DEGs of HIV chronic progressors compared with healthy donors, and ranked the top 100 nodes by degree (Fig. 2a). We found that most of these 100 nodes were upregulated interferon-stimulated genes (ISGs) such as *STAT1* (degree = 86), *IRF7* (degree = 55), *ISG15* (degree = 51), *MX1* (degree = 45), *GBP1* (degree = 44), *OAS1* (degree = 41), *IFIT3* (degree = 38), *IFIT1* (degree = 37) and *IFI44L* (degree = 35) [22, 36–38].

We identified 92 nodes from the PPI network of the CD8^+^ T cell-specific DEGs of HCV chronic progressors compared to healthy donors **(**Fig. 2b). The following eight HCV DEG hub nodes ranked most highly, including *IL1*-*β* (degree = 31), *CXCL8* (also known as *IL*-*8* and degree = 31), *TLR2* (degree = 28), *IL1RN* (degree = 21), *TREM1* (degree = 19), *CXCL2* (degree = 18), *PTGS2* (degree = 17) and *FPR1* (degree = 17). Of these, *CXCL8*, *TLR2* and *FPR1* are also upregulated ISGs [39].

We further investigated whether some of the CD8^+^ T cell DEGs were shared in mono-HIV and HCV chronic progressors compared with healthy donors. The Venn diagram showed that 17 DEGs were significantly altered (Fig. 2c and Table 2). GO analysis was carried out for the functional investigation of 17 co-DEGs, revealing that the “cellular defense response” term was significantly enriched (P = 0.043).

**Table 2:** Information regarding the 17 co-DEGs

	Gene symbol	Entrez ID	Official full name	|Log FC|
1	SPRY1	10252	Sprouty RTK signaling antagonist 1	0.7262
2	CCNG2	901	Cyclin G2	0.6149
3	CEBPD	1052	CCAAT enhancer binding protein delta	0.7097
4	NCR3	259197	Natural cytotoxicity triggering receptor 3	0.6289
5	LDLR	3949	Low density lipoprotein receptor	0.6821
6	SCRN1	9805	Secernin 1	0.7670
7	ZBTB16	7704	Zinc finger and BTB domain containing 16	0.6709
8	CCR5	1234	C–C motif chemokine receptor 5 (gene/pseudogene)	1.3274
9	TOX	9760	Thymocyte selection associated high mobility group box	0.7474
10	PRF1	5551	Perforin 1	1.0301
11	AGAP1	116987	ArfGAP with GTPase domain, ankyrin repeat and PH domain 1	0.6469
12	SPATS2L	26010	Spermatogenesis associated serine rich 2 like	1.1866
13	CAMK2N1	55450	Calcium/calmodulin dependent protein kinase II inhibitor 1	0.7255
14	CAST	831	Calpastatin	0.5967
15	CFAP20	29105	Cilia and flagella associated protein 20	0.5973
16	KLRB1	3820	Killer cell lectin like receptor B1	1.5773
17	C6orf48	50854	Chromosome 6 open reading frame 48	0.7269

### Functional GO terms and pathway enrichment analysis of DEGs in the CD8^+^ T cells of mono-HIV and HCV chronic progressors compared with healthy donors

**Fig. 3:**
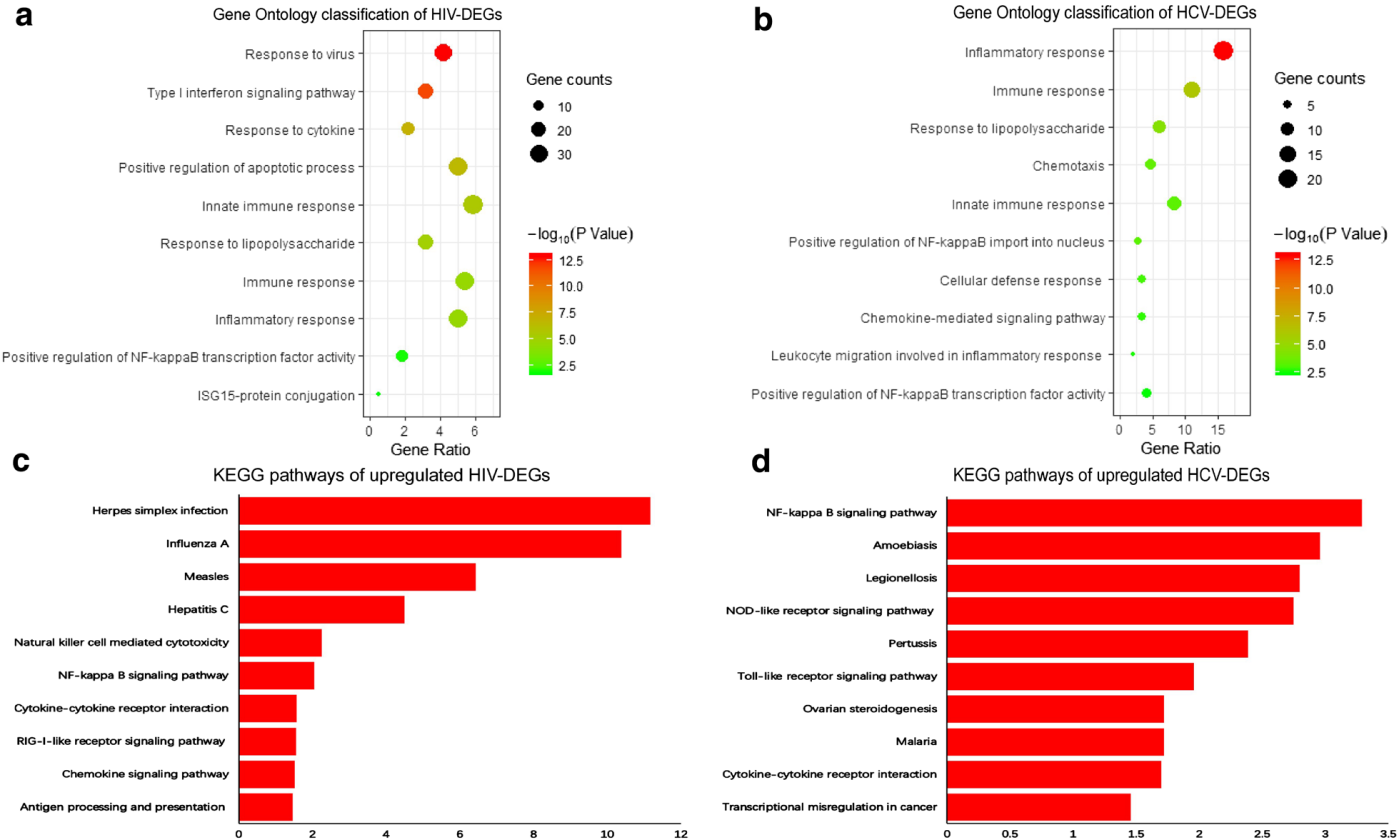
GO and KEGG analysis of DEGs. **a** Gene Ontology (GO) classification of DEGs from HIV chronic progressors compared with healthy donors. **b** GO classification of DEGs from HCV chronic progressors compared with healthy donors. **c** KEGG pathways of upregulated DEGs from HIV chronic progressors compared with healthy donors. **d** KEGG pathways of upregulated DEGs from HCV chronic progressors compared with healthy donors

GO and KEGG pathway analysis were carried out to investigate the common biological processes and pathways associated with the CD8^+^ T cells DEGs after mono-HIV and HCV infection. Following GO analysis, the “innate immune response”, “immune response”, “inflammatory response”, “positive regulation of NF-kappa B transcription factor activity” and “response to lipopolysaccharide” were significantly enriched for DEGs in the CD8^+^ T cells of both HIV (Fig. 3a) and HCV chronic progressors (Fig. 3b), compared with healthy donors.

In the pathway analysis, we identified significantly upregulated pathways enriched for DEGs in the CD8^+^ T cells from mono-HIV (Fig. 3c) and HCV chronic progressors (Fig. 3d), compared with healthy donors. The “NF-kappa B signaling pathway” and “cytokine–cytokine receptor interaction signaling pathway” were significantly upregulated in CD8^+^ T cells after mono-HIV and HCV infection. In addition, “Toll-like receptor signaling pathway” (P = 0.078 in HIV infection, P = 0.011 in HCV infection) and “TNF signaling pathway” (P = 0.08 in HIV infection, P = 0.079 in HCV infection) also have upregulated tendency after mono-HIV and HCV infection.

### DEGs from HIV non-progressors and HCV resolvers compared with corresponding chronic progressors and associated pathway enrichment analysis

Next, in order to understand the changes experienced by CD8^+^ T cells at different stages of disease progression, we compared the transcriptional profiles of CD8^+^ T cell from HIV non-progressors and HCV resolvers, versus chronic progressors. At first, we identified 92 DEGs by comparing the transcriptional profiles of CD8^+^ T cell from HIV non-progressors versus chronic progressors. Of the 92 DEGs, 47 genes were upregulated and 45 genes were downregulated (Additional file 4). Then, we identified 50 DEGs by comparing the transcriptional profiles of CD8^+^ T cell from HCV resolvers versus chronic progressors. Among the 50 DEGs, 13 genes were upregulated and 37 genes were downregulated (Additional file 5).

**Fig. 4:**
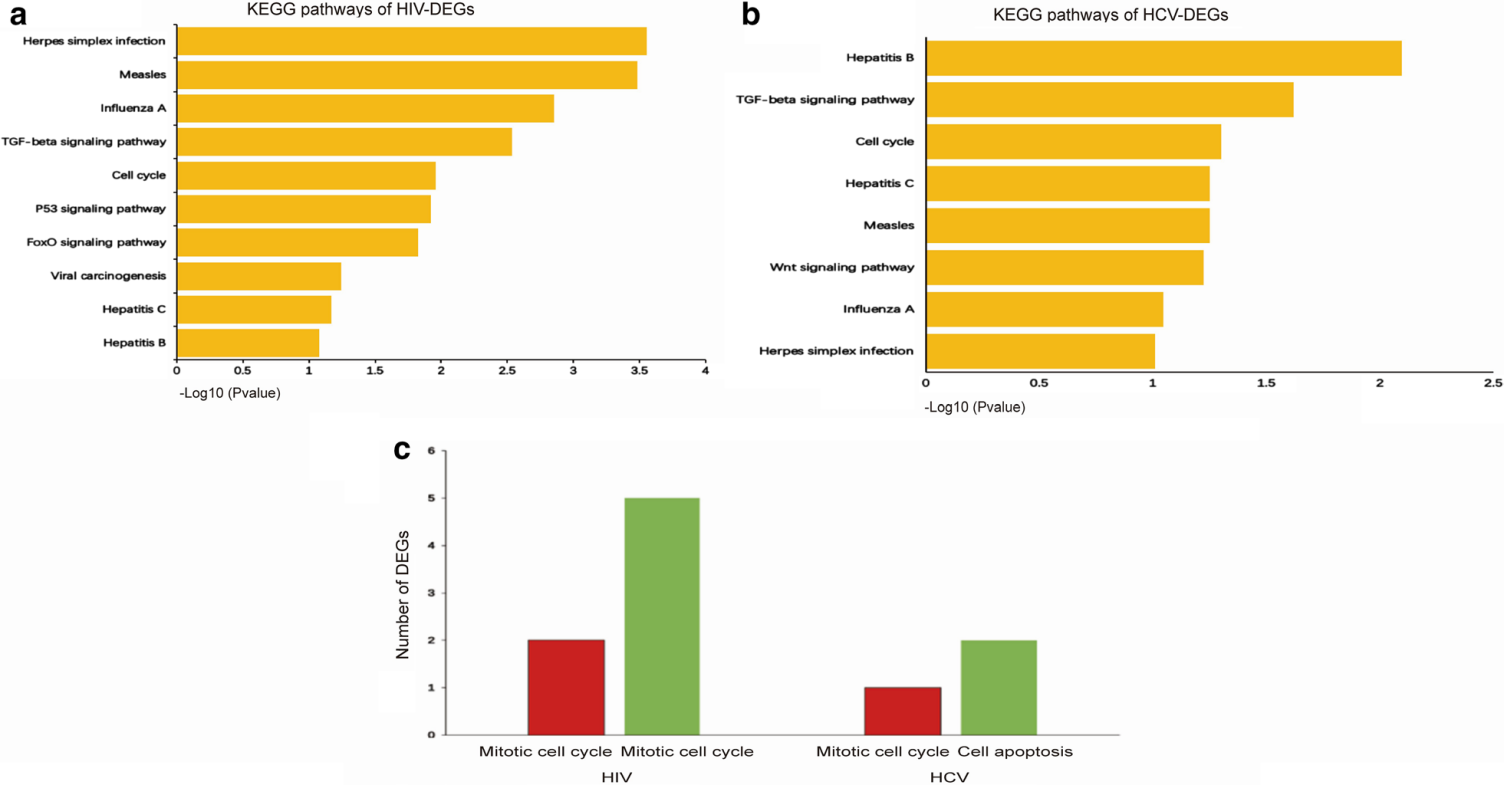
KEGG pathways of DEGs. **a** KEGG pathways of DEGs from HIV non-progressors compared with chronic progressors. **b** KEGG pathways of DEGs from HCV resolvers compared with chronic progressors. **c** Number of DEGs identified in the CD8^+^ T cells of HIV non-progressors and HCV resolvers compared with chronic progressors, involved in the mitotic cell cycle and cell apoptosis processes. Red = upregulated DEGs, green = downregulated DEGs

KEGG pathway analysis was subsequently carried out for the functional investigation of DEGs in the CD8^+^ T cell of HIV non-progressors and HCV resolvers, versus the corresponding chronic progressors. Pathway analysis identified that 7 pathways were commonly shared in HIV non-progressors (Fig. 4a) and HCV resolves (Fig. 4b), compared to chronic progressors. This included the “TGF-beta signaling”, “cell cycle”, “herpes simplex infection”, “hepatitis B”, “hepatitis C”, “measles” and “influenza A” pathways.

Cell cycle is associated with CD8^+^ T cell function during viral infection. We then performed the GO cell cycle analysis with the upregulated and downregulated DEGs to investigate the alterations of cell cycle. In HIV non-progressors versus chronic progressors, 5 downregulated genes and 2 upregulated genes were shown to be involved in the mitotic cell cycle, with all 7 genes being essential for mitosis. Upregulated genes included *PLK2* and *TUBGCP3* and downregulated genes comprised *HSPA1A*, *CCNA2*, *PSME2*, *TOP2A* and *NDC80*. The larger number of downregulated genes compared to upregulated genes may be indicative of mitosis attenuation. In HCV resolvers versus chronic progressors, one upregulated gene, *RBL2*, was identified. RBL2 acts as a negatively regulated transcription factor of the mitotic cell cycle, and its expression may also be responsible for dampening cell division. In addition, we found two downregulated genes, *STAT5A* and *MTCH1*, associated with the cell apoptosis. However, no upregulated genes associated with cell apoptosis were found in the CD8^+^ T cells of HCV resolvers versus chronic progressors (Fig. 4c). The above results suggest that in HIV non-progressors and HCV resolvers, disease progression may be prevented by increased CD8^+^ T cell survival, mediated by a combination of an attenuated mitotic cell cycle and reduced apoptosis.

### PPI network analysis of CD8^+^ T cell DEGs of HIV non-progressors and HCV resolvers, compared with chronic progressors, and the identification of predicted miRNAs

Finally, we performed PPI network analysis of DEGs in the CD8^+^ T cells of HIV non-progressors and HCV resolvers, versus the corresponding chronic progressors, with the aim of identifying hub nodes. Given the multi-targeting property of miRNA, we expected to identify miRNAs targeting both HIV and HCV hub genes to intervene in HIV and HCV mono- and co-infection.

**Fig. 5:**
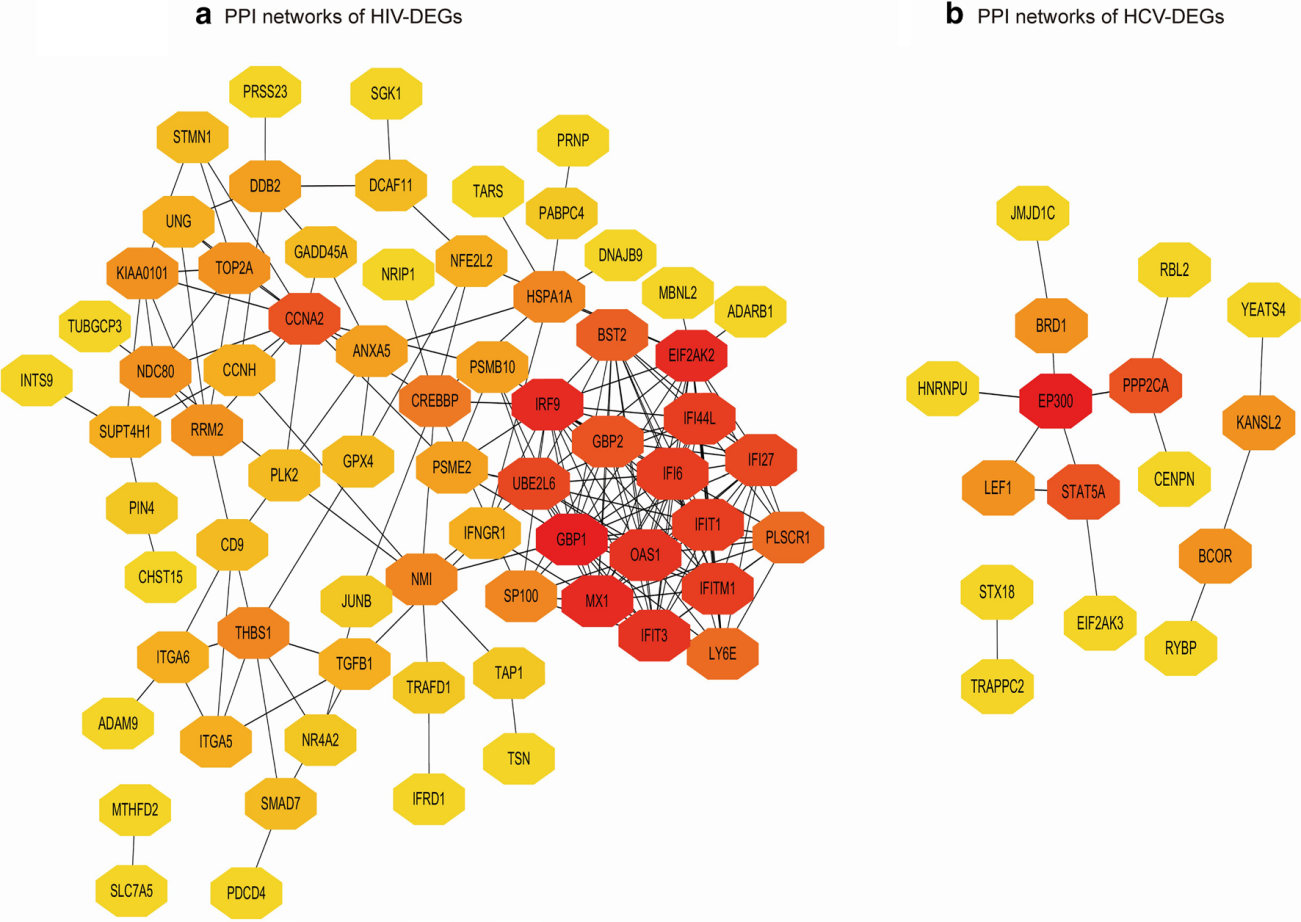
PPI networks. **a** PPI network of DEGs from HIV non-progressors compared with chronic progressors. **b** PPI network of DEGs from HCV resolvers compared with chronic progressors. In **a** and **b** red = greater degree, yellow = lesser degree

In HIV non-progressors versus chronic progressors, we identified 10 hub nodes, including *GBP1* (degree = 19), *MX1* (degree = 17), *IRF9* (degree = 17), *EIF2AK2* (degree = 17), *IFIT3* (degree = 16), *OAS1* (degree = 16), *IFI44L* (degree = 15), *IFIT1* (degree = 15), *IFI6* (degree = 15) and *IFITM1* (degree = 15) (Fig. 5a). Most of the listed hub nodes were downregulated ISGs. In HCV resolvers versus chronic progressors, we identified three hub nodes, including *EP300* (degree = 5), *STAT5A* (degree = 3) and *PPP2CA* (degree = 3), all of which were downregulated (Fig. 5b).

In order to interfere with disease progression by identifying key miRNAs with the capacity to target both HIV and HCV hub genes, we performed prediction analysis using mirDIP and miRDB bioinformatic tools. We identified 9 candidate miRNAs targeting *IFIT3* in the context of HIV infection and 16 candidate miRNAs targeting *STAT5A* in HCV infection (Additional file 6). We found the miR-143-3p could target both *IFIT3* and *STAT5A*. Clapé et al. evidenced that *ERK5* is a miR-143 target gene [40], whilst, Wilhelmsen et al. demonstrated that this gene plays a proinflammatory role in primary human endothelial cells and monocytes [41]. We thus deduced that miRNA-143-3p may exert its anti-inflammatory effects by suppressing *ERK5*. Finally, through cell transfection and PCR technology, we found that overexpression of miRNA-143-3p can suppress ERK5 expression in primary CD8^+^ T cells by paired t-test, suggesting the fine tune effect of miRNA-143-3p on ERK5 (Additional file 7).

## Discussion

Identification of the most relevant genes and pathways involved in mono-HIV and HCV infection is important for broadening our understanding of the molecular and cellular processes determining disease progression. In our study, we first identified CD8^+^ T cell-associated DEGs in mono-HIV and HCV chronic progressors, compared with healthy donors, and found that ISGs were commonly upregulated. Furthermore, we found that the “cytokine–cytokine receptor interaction” and “NF-kappa B” signaling pathways were also upregulated after mono-HIV and HCV infection. In addition, we observed attenuated mitosis and reduced ISG expression in the CD8^+^ T cells of HIV non-progressors and HCV resolvers, compared with chronic progressors. Finally, we identified and predicted miRNAs that may interfere with HIV and HCV mono- and co-infection.

Firstly, the comparison of CD8^+^ T cell transcriptional profiles after mono-HIV and HCV infection demonstrated that certain ISGs, including *STAT1*, *ISG15*, *IFIT1*, *IFIT3* and *IFI44L*, were significantly upregulated in HIV chronic progressors. Meanwhile, *CXCL8*, *TLR2* and *FPR1* were significantly upregulated in HCV chronic progressors. Several studies have highlighted the role of ISGs in the progression of mono-HIV and HCV infection [22, 42, 43]. Type-I interferons (IFNs) are of critical importance in the control of viral disease due to their potent antiviral effects, mediated by interferon-induced proteins [44, 45]. However, it is also well established that type-I IFNs are especially effective at very low concentrations and their expression is required locally [46, 47]. Emerging lines of evidence reveal that high and sustained levels of type-I IFN expression are associated with hyper-immune activation and disease progression in persistent infection [47–51]. The upregulated “cytokine–cytokine receptor” and “NF-kappa B” signaling pathways also reflects a high level of immune activation [52–54]. The dynamics of type-I IFN expression distinguishes between simian immunodeficiency virus (SIV) infection of natural hosts, that do not develop AIDS, from pathogenic SIV infection [55–58]. Natural hosts can rapidly silence their type-I IFN response after acute SIV infection, whereas, disease-susceptible macaque species maintain type-I IFN signaling indefinitely, thus triggering the hyper-activation of the immune system and contributing to an environment that favors progression to AIDS [56–60]. Persistent hyper-immune activation also results in immune exhaustion [61–63], including the loss of CD8^+^ T cell function. Several studies have demonstrated that the hyper-activation of virus-specific T cells, caused by the strong and sustained production of type-I IFNs, may hamper viral clearance [50, 64]. In addition, an inefficient T cell response that fails to clear HCV infection creates a chronic inflammatory process, the end result of which are hepatic fibrosis, cirrhosis and HCC [65]. Collectively, our results indicate that the increased expression of ISGs, which leads to elevated levels of immune activation, is a key factor affecting disease progression.

Secondly, the comparison of CD8^+^ T cell transcriptional profiles of HIV non-progressors and HCV resolvers, versus chronic progressors, revealed several common pathways associated with both HIV and HCV, including the “herpes simplex infection” “measles”, “influenza A”, “hepatitis B”, “hepatitis C”, “TGF-beta” and “cell cycle” pathways. Maintaining normal cell cycle function is essential for the antiviral activity of CD8^+^ T cells. However, CD8^+^ T cell turnover is increased in HIV infected individuals, which reflects a hyper-activated immune status that contributes to the exhaustion and depletion of this important cell subset [66–69]. By comparing HIV non-progressors with chronic progressors, we found that all the DEGs associated with the mitotic cell cycle were essential for mitosis, and the majority of these DEGs were downregulated. The relatively high numbers of downregulated genes associated with the mitotic cell cycle may suggest CD8^+^ T cell mitotic attenuation in HIV non-processors compared with chronic progressors. By comparing HCV resolvers with chronic progressors, we found one upregulated gene, *RBL2*, a negatively regulated transcription factor involved in the mitotic cell cycle, which may also imply an attenuated CD8^+^ T cell mitosis. Additionally, we identified two downregulated cell apoptosis-associated genes, *STAT5A* and *MTCH1*, while no apoptotic genes were upregulated, which may be indicative of a reduction in the apoptosis of the CD8^+^ T cells within HCV resolvers, compared with chronic progressors. The relatively low turnover of CD8^+^ T cells in HIV non-progressors and HCV resolvers may be one reason for their ability to maintain the non-progression of disease.

Since miRNAs are a class of non-coding RNAs involved in the regulation of gene expression via mRNA degradation or translational repression [70–73], we expected to find miRNAs capable of interfering with HIV and HCV mono- and co-infection. Moreover, as no co-DEGs were found in HIV non-progressors and HCV resolvers, compared with chronic progressors, we used the PPI network to identify DEG hub nodes. The PPI analysis of HIV infection revealed that the identified hub genes were mostly downregulated ISGs, which directly opposed the above-mentioned result expected for the comparison of chronic progressors with healthy donors. Our PPI analysis of HCV infection showed that the hub gene *STAT5A*, responsible for type-I IFN signal transduction to the promoters of ISGs, was downregulated [74, 75]. Given the multi-targeting property of miRNAs [76–79], we finally identified the miR-143-3p, with the ability to target both *IFIT3* and *STAT5A,* in HIV and HCV infection, respectively. Clapé et al. evidenced that *ERK5* is a miR-143 target gene [40], whilst, Wilhelmsen and et al. demonstrated that this gene plays a proinflammatory role in primary human endothelial cells and monocytes [41]. We thus deduced that miRNA-143-3p may exert its anti-inflammatory effects by suppressing *ERK5*. Such miRNA-143-3p-mediated suppression of inflammatory cytokine overproduction may be exploited as a therapeutic strategy in the context of HIV/HCV mono- and co-infection.

## Conclusion

In summary, our analysis of microarray studies shows that the upregulation of ISGs and the NF-kappa B signaling pathway are collectively indicative of persistent immune activation in the context of mono-HIV and HCV infection. The decreased turnover of CD8^+^ T cells, as seen in HIV non-progressors and HCV resolvers, may reduce CD8^+^ T cell exhaustion and limit disease progression.

## Supplementary information

**Additional file 1.** The PCAs of the matrix before and after Combat normalization

**Additional file 2.** CD8^+^ T cell DEGs from HIV chronic progressors compared with healthy donors

**Additional file 3.** CD8^+^ T cell DEGs from HCV chronic progressors compared with healthy donors

**Additional file 4.**CD8^+^ T cell DEGs from HIV non-progressors compared with chronic progressors

**Additional file 5.** CD8^+^ T cell DEGs from HCV resolvers compared with chronic progressors

**Additional file 6.***IFIT3*- and *STAT5A*-targeting miRNAs

**Additional file 7.** The experiment of the effect of miRNA-143-3p on suppressing ERK5

## Data Availability

GSE49954, GSE24081, GSE6740, GSE93711 and GSE93712 datasets were downloaded from GEO (http://www.ncbi.nlm.nih.gov/geo/). R packages of “affy”, and “limma” (http://www.bioconductor.org/packages/release/bioc/html/affy.html), provided by a bioconductor project, were applied to assess GSE6740 and GSE49954 RAW datasets. INMEX program (http://www.inmex.ca) were applied to assess GSE24081, GSE6740, GSE93711 and GSE93712.

## Abbreviations

GEO: Gene Expression Omnibus
DEGs: Differentially expressed genes
INMEX: Integrative meta-analysis of expression data
PPI: Protein–protein interaction
GO: Gene Ontology
miRNAs: microRNAs
ISGs: Interferon-stimulated genes
HIV: Human immunodeficiency virus
HCV: Hepatitis C virus
LTNPs: Long-term non-progressors
ECs: Elite controllers
STRING: Search Tool for the Retrieval of Interacting Genes
KEGG: Kyoto Encyclopedia of Genes and Genomes
IFNs: Interferons
HD: Healthy donor
CP: Chronic progressor
NP: Non-progressor
RP: Resolver patient
CXCL8: C-X-C motif chemokine ligand 8
CXCL2: C-X-C motif chemokine ligand 2
STAT1: Signal transducer and activator of transcription 1
IRF7: Interferon regulatory factor 7
ISG15: Interferon-stimulated gene 15
MX1: MX dynamin like GTPase 1
GBP1: Guanylate binding protein 1
OAS1: 2′-5′-oligoadenylate synthetase 1
IFIT3: Interferon induced protein with tetratricopeptide repeats 3
IFIT1: Interferon induced protein with tetratricopeptide repeats 1
IFI44L: Interferon induced protein 44 like
IL1-β: Interleukin 1 beta
TLR2: Toll like receptor 2
IL1RN: Interleukin 1 receptor antagonist
TREM1: Triggering receptor expressed on myeloid cells 1
PTGS2: Prostaglandin-endoperoxide synthase 2
FPR1: Formyl peptide receptor 1
PLK2: Polo like kinase 2
TUBGCP3: Tubulin gamma complex associated protein 3
HSPA1A: Heat shock protein family A (Hsp70) member 1A
CCNA2: Cyclin A2
PSME2: Proteasome activator subunit 2
TOP2A: DNA topoisomerase II alpha
NDC80: NDC80 kinetochore complex component
RBL2: RB transcriptional corepressor like 2
STAT5A: Signal transducer and activator of transcription 5A
MTCH1: Mitochondrial carrier 1
IRF9: Interferon regulatory factor 9
EIF2AK2: Eukaryotic translation initiation factor 2 alpha kinase 2
IFI6: Interferon alpha inducible protein 6
IFITM1: Interferon induced transmembrane protein 1
EP300: E1A binding protein p300
PPP2CA: Protein phosphatase 2 catalytic subunit alpha
