# Supplementary material for: Transcriptional insights into the CD8+ T cell response in mono-HIV and HCV infection
Source: J Transl Med. 2020 Feb 24;18:96. doi: 10.1186/s12967-020-02252-9 (PMC7038596; doi:10.1186/s12967-020-02252-9)
Supplement: Supplementary file 1 — Additional file 1. The PCAs of the matrix before and after Combat normalization [file 12967_2020_2252_MOESM1_ESM.docx]

**PCA plot of the HIV matrix before Combat normalization.**


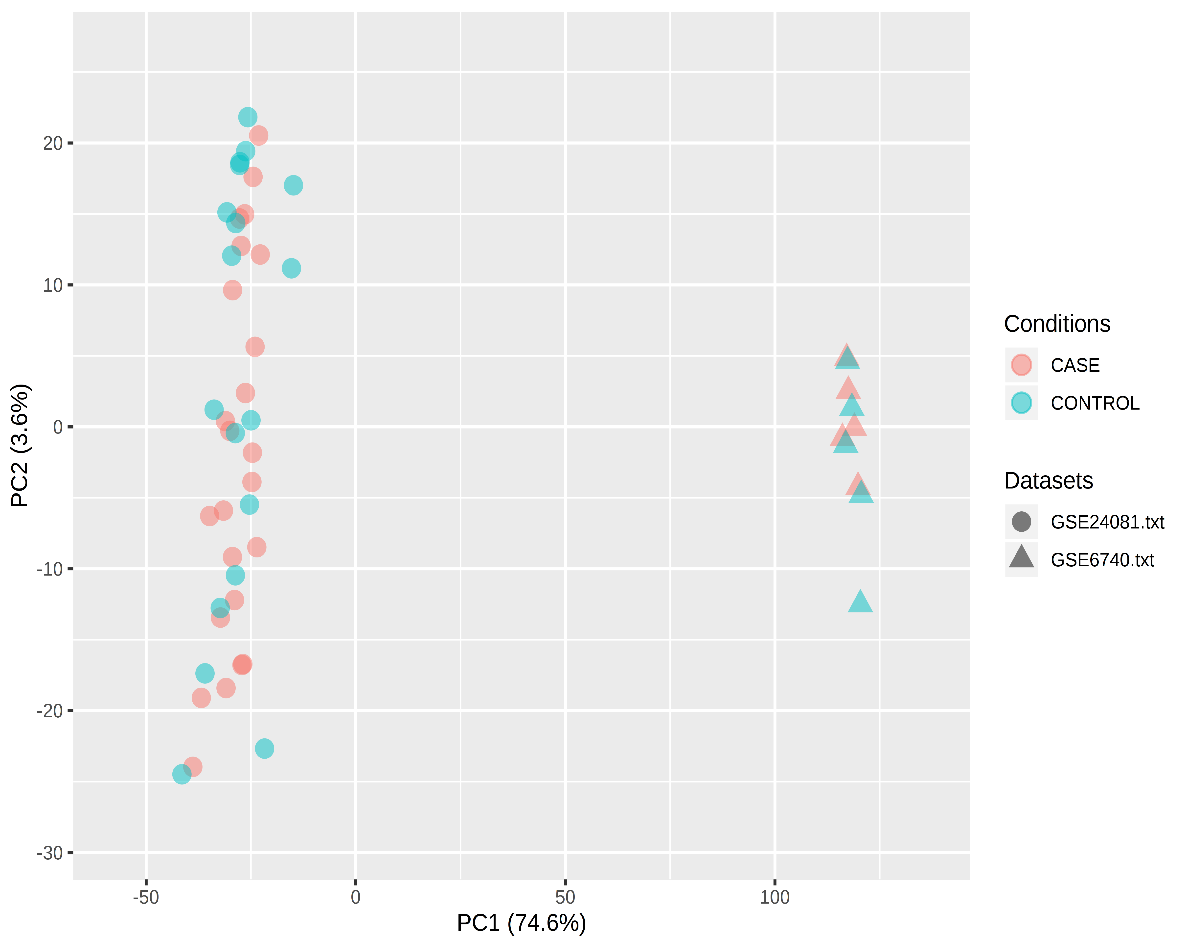


**PCA plot of the HIV matrix after Combat normalization.**

**
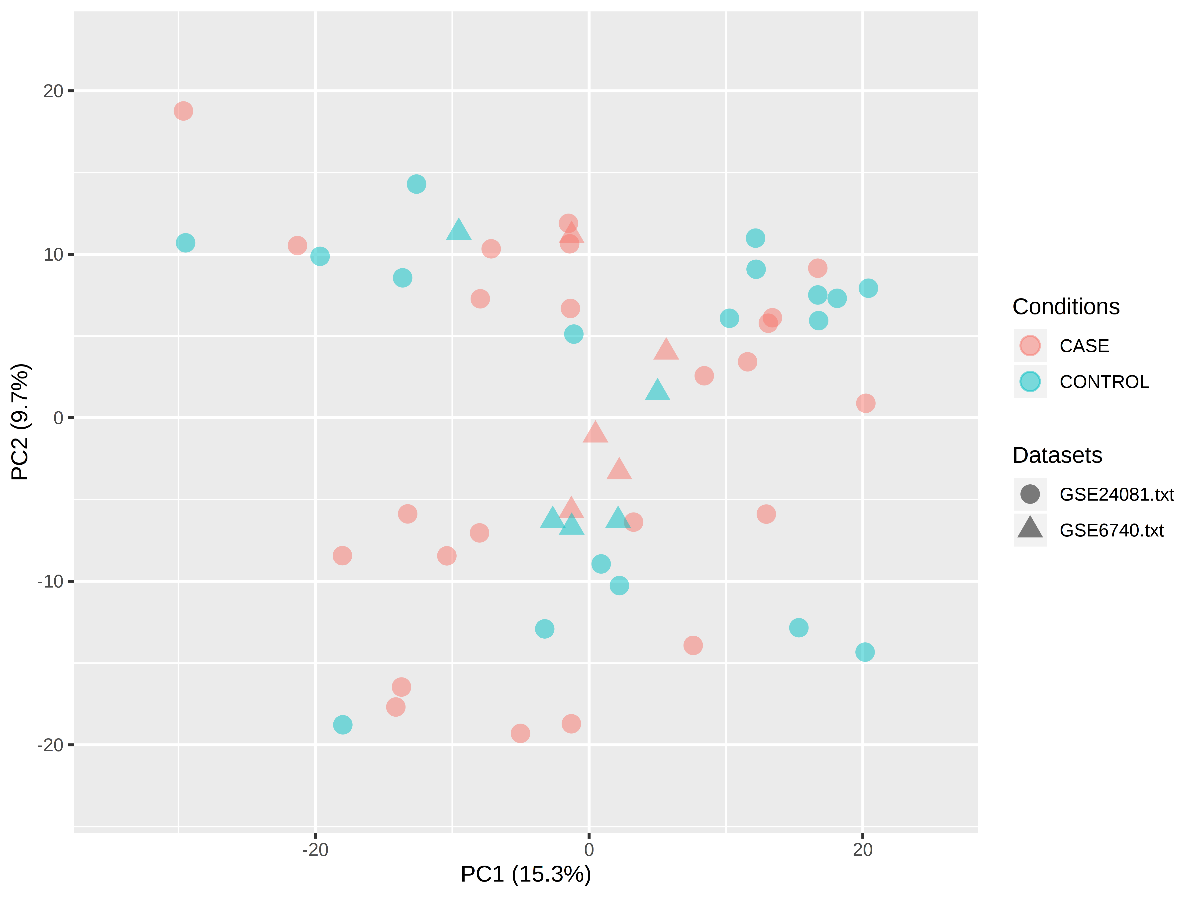
**

**PCA plot of the HCV matrix before Combat normalization.**

**
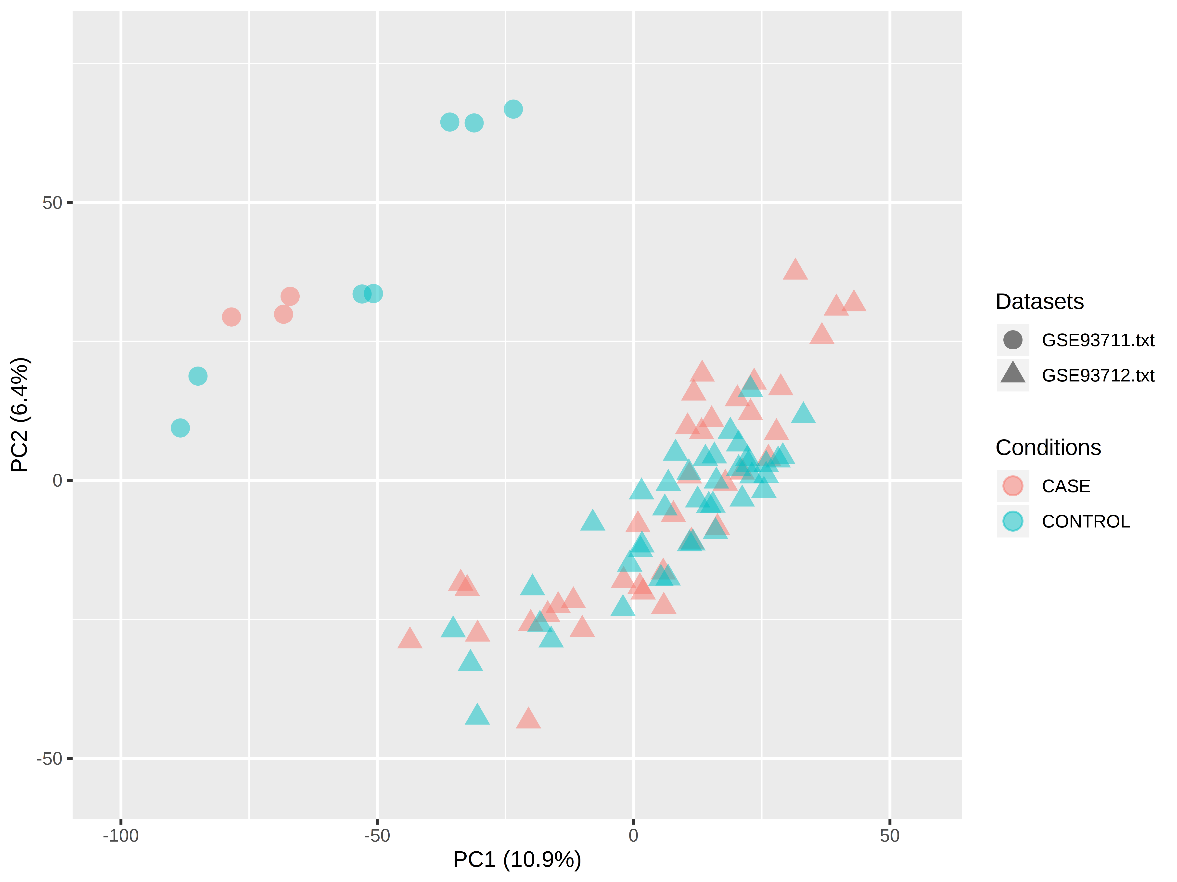
**

**PCA plot of the HCV matrix after Combat normalization.**

**
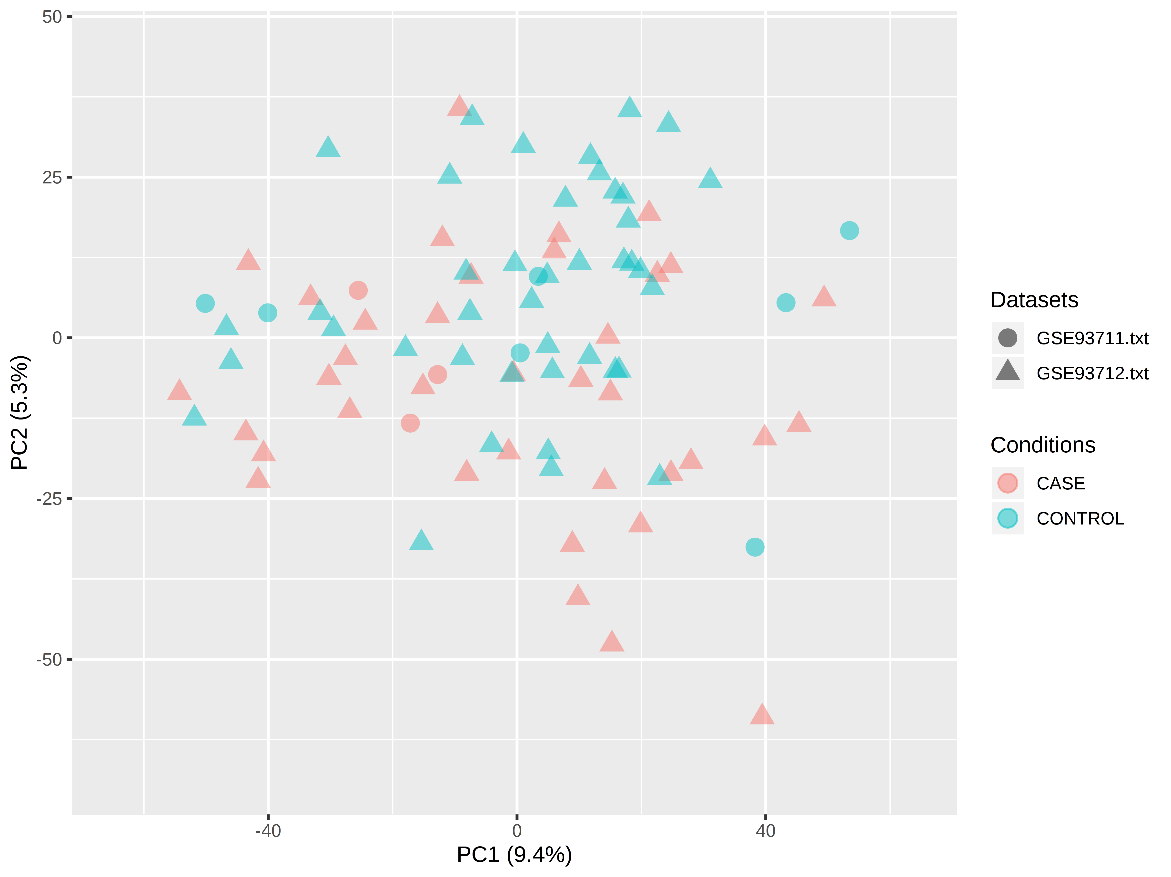
**
