# Supplementary material for: Transcriptional insights into the CD8+ T cell response in mono-HIV and HCV infection
Source: J Transl Med. 2020 Feb 24;18:96. doi: 10.1186/s12967-020-02252-9 (PMC7038596; doi:10.1186/s12967-020-02252-9)

**The suppression of ERK5 mRNA expression achieved by transfection of miRNA-143-3p to CD8^+^T cells.**

**Materials and Methods**

**Study Population**

Samples were obtained from four healthy donors (age, mean 25.5 years; gender, three males and one female) and all healthy donors signed informed consents.

**Isolation of Cells**

PBMCs were obtained by Ficoll–Hypaque density gradient centrifugation. If indicated, CD8+ T cells were further purified from isolated PBMCs by negative selection with magnetic beads using a CD8+T cell Isolation Kit (Stem Cell Technologies, Vancouver, Canada).

**Cell Culture**

The CD8+ T cells were maintained in RPMI1640 media (HyClone, Logan, UT, USA) supplemented with 10% fetal bovine serum.

**Transfection**

Transfection of miRNA-143-3p to isolated CD8+T cell from healthy donors was achieved using Lipofectamine® RNAiMAX (Invitrogen). 20 μM miRNA-143-3p mimics or controls (GenePharma, Shanghai, China) were transfected to CD8+T cells according to the protocol provided by the manufacturer.

**RT-PCR Quantification of miRNA and mRNA**

We extracted miRNAs from cells using the miRNeasy Micro kit (Qiagen, Hilden, Germany). The RNA was reverse transcribed using a Primpscript® RT reagent kit (TAKARA, Dalian, China) according to the instructions provided by the manufacturer. Subsequently, RT-PCR was performed using a SYBR® Premix Ex Taq™ II (TAKARA). The levels of miRNA were normalized to the U6 small nucleolar RNA and quantified through the relative quantification method (2−ΔΔCt). Cellular total mRNA was isolated using the RNeasy Micro kit (Qiagen). The cDNA was generated using the Primpscript® RT reagent kit (TAKARA). The levels of mRNA were quantified through the SYBR® Premix Ex Taq™ II (TAKARA), normalized to GAPDH transcripts, and expressed using the relative quantification method (2−ΔΔCt).

**Statistical Analysis**

A paired t-test was used to analyze ERK5 mRNA expression achieved by transfection of miRNA-143-3p to CD8+T cells. Data analysis was performed using the GraphPad Prism Version 5.0 software packages. A P < 0.05 was considered statistically significant.

**Results.**

The suppression of ERK5 mRNA expression achieved by transfection of miRNA-143-3p to CD8^+^T cells.


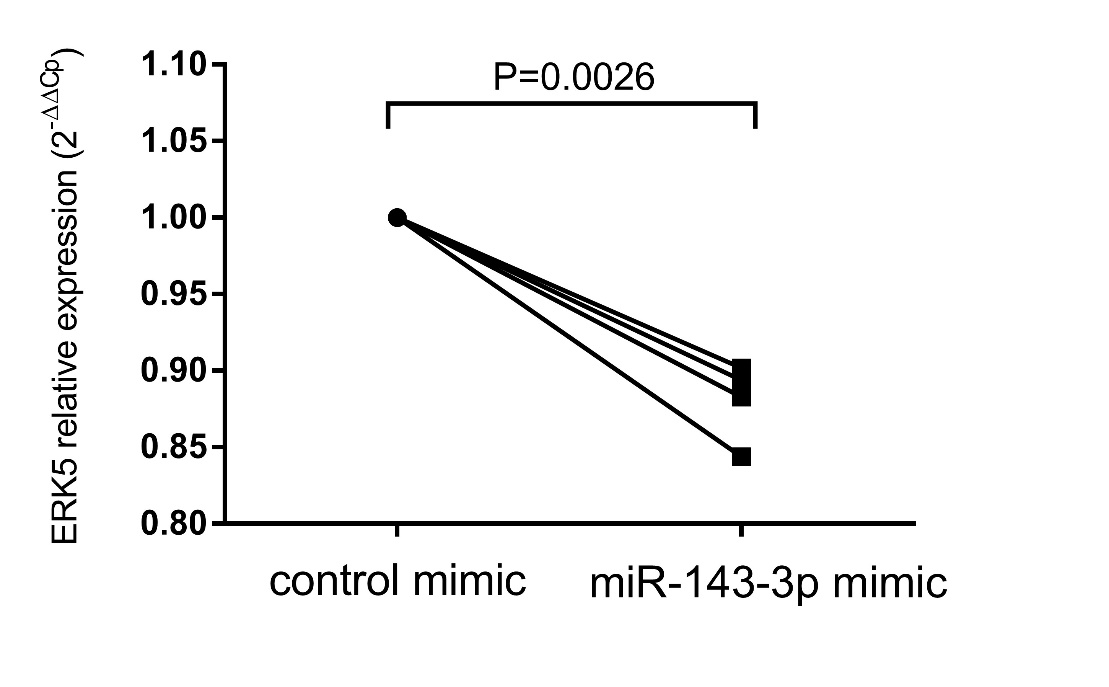

Supplement: Supplementary file 7 — Additional file 7. The experiment of the effect of miRNA-143-3p on suppressing ERK5 [file 12967_2020_2252_MOESM7_ESM.docx]
